# Treacle is Upregulated in Cancer and Correlates With Poor Prognosis

**DOI:** 10.3389/fcell.2022.918544

**Published:** 2022-06-20

**Authors:** Kezia Catharina Oxe, Dorthe Helena Larsen

**Affiliations:** Nucleolar Stress and Disease Group, Danish Cancer Society Research Center, Danish Cancer Society, Copenhagen, Denmark

**Keywords:** cancer, ribosome biogenesis, DNA damage response, chromatin, nucleolus, rDNA, treacle, TCOF1

## Abstract

Treacle/TCOF1 is an adaptor protein specifically associated with nucleolar chromatin. In the nucleolus it stimulates ribosome biogenesis, thereby promoting growth and proliferation. A second role of Treacle has emerged as a coordinator of the nucleolar responses to DNA damage, where it facilitates nucleolar DNA repair and cellular survival after genotoxic insults. The involvement of Treacle in multiple fundamental processes such as growth, proliferation, and genome stability, which are tightly linked to cancer, raises the question of Treacle’s role in the development of this disease. On one hand, overexpression of Treacle could stimulate nucleolar transcription and ribosome biogenesis providing a growth advantage in cancer cells. On the other hand, the function of Treacle as a gatekeeper in response to nucleolar DNA damage could favor mutations that would impair its function. In this perspective, we analyze paired Treacle expression data from the Cancer Genome Atlas (TCGA) and correlate expression with patient survival in different cancer types. We also discuss other recently published observations of relevance to the role of Treacle in cancer. In light of these new observations, we propose possible roles of Treacle in carcinogenesis and discuss its potential as a therapeutic target.

## 1 Introduction

Cancer is one of the leading causes of death worldwide, accounting for nearly 10 million deaths every year (Sung et al., 2021). The current situation calls for better prevention and new treatment options. In the last decade, tailored treatments for patients have developed rapidly with biomarkers being an important tool to determine tumor characteristics, and enable optimal matching of patient and treatment. In this perspective, we perform expression and survival analysis of the nucleolar protein Treacle to assess its potential as a biomarker in cancer.

The nucleolus is a membrane-less nuclear compartment responsible for the production of ribosomes and therefore indirectly regulating protein translation, growth, and proliferation (Derenzini et al., 2017). It is formed around a genomic region composed of hundreds of identical ribosomal RNA genes (rDNA), enabling the nucleolus to meet the cellular demand for proteins (McStay and Grummt, 2008; Drygin et al., 2010). The rDNA is transcribed in the nucleolus by RNA polymerase I (Pol I) and a large number of co-factors, producing rRNA that associates with ribosomal proteins to generate 1–2 million ribosomes per cell generation (Drygin et al., 2010; Correll et al., 2019). rDNA is the most transcribed region in the genome and can account for more than half of the active transcription in proliferating cells (Zylber and Penman, 1971; Warner, 1999). Growth stimulating pathways such as mTOR, PI3K/AKT, and MAPK/ERK also stimulate ribosome biogenesis (Stefanovsky et al., 2006; Gentilella et al., 2015; Derenzini et al., 2017) to enable cell growth.

Abrogation of nucleolar activity, however, can induce cell cycle arrest and cell death. Upon perturbation of ribosome biogenesis, the nucleolus releases ribosomal and tumor suppressor proteins to activate checkpoints (Grummt, 2013). The most studied example is the release of the ribosomal proteins RPL5/uL18 and RPL11/uL5 that form a complex with the 5S rRNA and sequester the ubiquitin ligase MDM2, resulting in stabilization of the tumor suppressor p53 (Bursać et al., 2012; Fumagalli et al., 2012; Sloan et al., 2013). The activation of p53 can lead to either cell cycle arrest or induction of cell death, making the nucleolus a key regulator of cell growth and proliferation.

Treacle, encoded by the TCOF1 gene, is a nucleolar chromatin-associated protein that promotes rDNA transcription and processing of rRNA (Valdez et al., 2004). First identified as a ribosome biogenesis factor, Treacle binds Upstream Binding Factor (UBF) and Pol I to initiate rDNA transcription. Treacle down-regulation impairs localization of UBF and Pol I to nucleolar chromatin, resulting in decreased rRNA transcription (Lin and Yeh, 2009). In addition, Treacle regulates rRNA processing by promoting 2′-O-methylation of rRNA (Gonzales et al., 2005).

rDNA is the most frequently rearranged genomic region and represents several challenges in relation to DNA repair (Stults et al., 2008). Its repetitive nature with identical genes placed on the five acrocentric chromosomes (13, 14, 15, 21 and 22) makes it vulnerable to faulty DNA repair (Potapova et al., 2019). Furthermore, the high level of transcription can potentially interfere with replication and is therefore a potent source of DNA damage (García-Muse and Aguilera, 2016). These features highlight the need for continuous maintenance of rDNA.

Recent studies have revealed that Treacle is also a central coordinator of the nucleolar response to multiple types of DNA damage and is important to uphold the integrity of rDNA. Double-strand breaks (DSBs) are a particularly harmful type of lesion as they can lead to mutations or deletions. Upon DSBs in the rDNA, Treacle is phosphorylated in an ATM-dependent manner and subsequently serves as a recruitment mediator for the MRE11-RAD50-NBS1 (MRN) complex (Korsholm et al., 2019) and TOPBP1 to DSB-associated chromatin regions in rDNA (Mooser et al., 2020). ATM-Treacle-MRN and TOPBP1 accumulation leads to ATR activation and a strong inhibition of rDNA transcription. Sustained DSB-signaling in rDNA leads to nucleolar reorganization and translocation of the damaged rDNA to nucleolar caps (Harding et al., 2015; van Sluis and McStay, 2015; Warmerdam et al., 2016; Korsholm et al., 2019; Marnef et al., 2019; Mooser et al., 2020). In nucleolar caps, homology-dependent rDNA repair takes place after separation of individual chromosomes into distinct caps (van Sluis and McStay, 2015; Korsholm et al., 2020). Treacle is characterized as a low-complexity protein, however details of the known structural-functional relationship was recently reviewed by Gál et al., 2022.

From the two well-characterized roles of Treacle it is clear that dysregulation of Treacle could play a role in cancer. Enlarged nucleoli and aberrant nucleolar morphology were linked to cancer over a century ago (Pianese, 1896; Derenzini et al., 1998). More recently, an upregulation of nucleolar activity has been found in almost all cancer types and addiction of cancer cells to ribosome biogenesis has been documented (Derenzini et al., 1998, 2000; Bywater et al., 2012; Carotenuto et al., 2019). Cancer cells need increased nucleolar activity to sustain rapid proliferation, suppress stress signaling and checkpoint activation, and to evade cell death (Bywater et al., 2012). It would therefore be reasonable to assume that upregulation of Treacle can promote cancer. However, it is also a central coordinator of the nucleolar responses to DNA damage (Korsholm et al., 2019, 2020; Mooser et al., 2020), and mutations in DSB-repair proteins are frequent in cancer (Aparicio et al., 2014). Such mutations are needed for cancer cells to escape checkpoint control and to acquire genomic instability, thereby fueling tumor development. Altogether, this poses the question, if and how Treacle supports cancer cells, and how an altered status of Treacle in cancer correlates with cancer prognosis and survival.

### 2 Treacle is Overexpressed in Multiple Cancer Types

**FIGURE 1:**
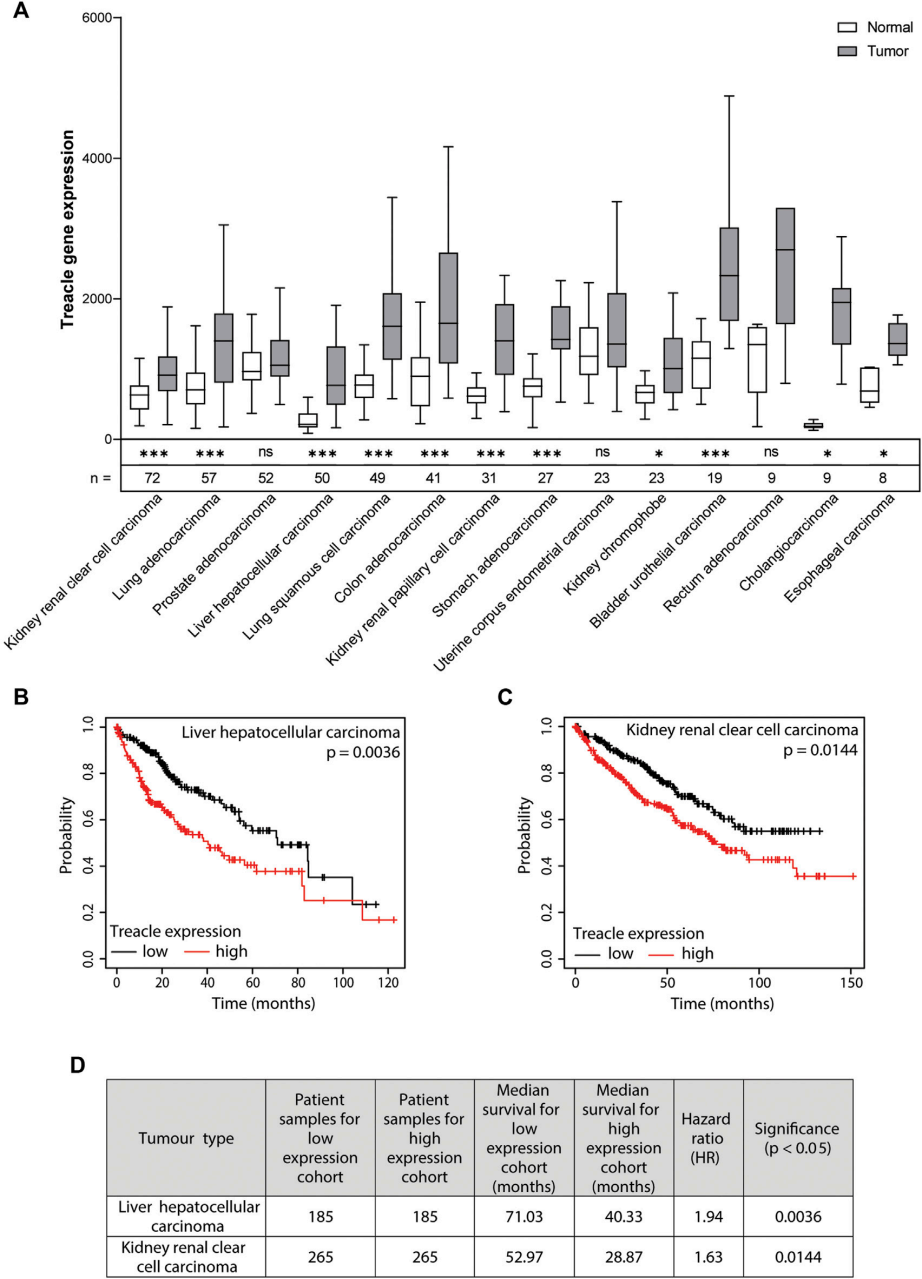
Increased Treacle expression in cancer correlates with poor survival. **(A)** TNM plot: differential gene expression analysis of Treacle RNA-Seq data in paired normal and tumor tissue across anatomical sites (https://tnmplot.com/analysis/) (Bartha and Győrffy, 2021). Significance of the difference in Treacle expression levels between paired normal and tumor tissue was estimated by TNM plot using a Mann-Whitney U test and the resulting p-values were FDR-adjusted prior to assessment of significant correlations [*p* < 0.05 (* > 0.05, ** > 0.01, *** > 0.001)]. **(B, C)** Kaplan-Meier survival analysis with stratification according to expression levels of Treacle based on the median cut-off between the lower and upper quartile (https://kmplot.com/analysis/index.php?p=service&cancer=pancancer_rnaseq) (Nagy et al., 2021). Cox proportional hazards ratio (HR) analysis was utilized to assess the correlation between Treacle gene expression and overall survival. Two cancer types with significant correlation (FDR-adjusted p-values; *p* < 0.05) between high Treacle expression levels and reduced overall survival were found: **(B)** liver hepatocellular carcinoma (HR = 1.94, p = 0.0036) and **(C)** kidney renal clear cell carcinoma (HR = 1.63, *p* = 0.0144). **(D)** Overview of low and high cohort sample sizes, median survival, HR, and FDR-adjusted significance (*p* < 0.05) for liver hepatocellular carcinoma and kidney renal clear cell carcinoma.

To understand if Treacle expression is altered across different cancers, we analyzed RNA-Seq data from TCGA using the TNM plot software (Bartha and Győrffy, 2021). We used Treacle expression data from paired normal and adjacent tumor samples from 470 patients. For our analysis, we included cancer types with available data from a minimum of five patients. Interestingly, we found significantly higher Treacle expression levels in 11 out of 14 cancers (*p* < 0.05 following False Discovery Rate (FDR) adjustment by Bonferroni correction (Benjamini and Hochberg, 1995) of the obtained *p*-values from TNM plot) (Figure 1A). Rectum adenocarcinoma tumor samples also have higher expression levels compared to normal, however, the difference was not significant. In prostate adenocarcinoma and uterine corpus endometrial carcinoma expression levels were comparable in paired normal and tumor samples. In summary, these results suggest that Treacle expression becomes upregulated in most cancers.

From the prevalent upregulation of Treacle across several cancer types, we wanted to analyze whether there is a correlation between high Treacle expression levels and patient survival. We analyzed pan-cancer RNA-Seq data by use of a Kaplan-Meier Plotter analyzing clinically available data from 7489 patients with 18 different tumor types obtained from TCGA (Nagy et al., 2021). The patients were separated into low and high Treacle expression cohorts with a cut-off defined by the median. The extracted *p*-values from the Kaplan-Meier plotter were further FDR adjusted by Bonferroni correction (Benjamini and Hochberg, 1995). We found a significant correlation (*p* < 0.05) between increased Treacle expression levels and poor survival in liver and renal cancer (Figures 1B–D). Amongst the cancer types where the correlation was not statistically significant both elevated and reduced hazard ratios were found (data not shown).

The correlation between high Treacle expression levels and poor survival in liver and renal cancer was also found in the Human Protein Atlas online resource (Uhlén et al., 2015). Their analysis was also based on RNA-Seq data of Treacle expression from the TCGA database (Uhlén et al., 2015).

Several recent studies focusing on the prognostic and potential therapeutic value of Treacle in cancer reach conclusions in agreement with our data. Wu et al. proposed a role for Treacle in oncogenic activation and promotion of tumorigenesis in human hepatocellular carcinoma (HCC) (Wu et al., 2021). They found a positive correlation between Treacle expression levels and advanced pathological stages and histological grade in HCC, suggesting a role for Treacle in cancer progression. In addition, they found that high Treacle expression resulted in a significant decrease in overall survival probability in HCC patients (Wu et al., 2021). This data is in agreement with the RNA-seq data analyzed here, showing a significant upregulation of Treacle expression in liver cancer as well as a significant correlation between high Treacle expression and poor survival (Wu et al., 2021).

Another recent study by Hu et al. showed that Treacle mRNA is upregulated in 32% of triple negative breast cancers (TNBC) (Hu et al., 2022). The upregulation of Treacle was validated at the protein level in breast cancer cell lines and by immunohistochemistry in a cohort of breast cancer patients (Hu et al., 2022). Furthermore, the authors investigated the association between Treacle status and patient outcome and demonstrated that high levels of Treacle expression correlated with poor survival both in TNBC and across all breast cancers. In the case of TNBC patients, correlation between Treacle expression, tumor grade, and TNM stage was observed, pointing to a role of Treacle in advanced stages of cancer in TNBC patients (Hu et al., 2022).

An additional pan-cancer study by Gu et al. analyzed expression levels from un-paired normal and tumor samples and prognostic value of Treacle. In this study, the authors found Treacle expression levels to be significantly upregulated in a broad range of cancers (Gu et al., 2022). This study also correlated Treacle expression levels with patient outcome and found an overall correlation between high Treacle expression levels and poor survival in five cancer types. However, variation in the results were observed depending on whether the authors used the GEO or TCGA dataset, making it difficult to interpret the results.

In summary, our results and the conclusions from recent studies show an upregulation of Treacle in cancer and indicate that it has potential as a prognostic marker in a subset of cancers.

### 3 Treacle can Promote Tumor Initiation

Conditions where Treacle expression is compromised may provide valuable clues to understand its importance in cancer. A complete loss of Treacle does not seem to be compatible with life, but haploinsufficiency occurs upon mutations in Treacle and gives rise to Treacher Collins Syndrome (TCS) (Dixon et al., 2006; Lin and Yeh, 2009). TCS is a rare autosomal dominant craniofacial disorder (Fazen et al., 1967) strongly linked with p53-dependent cell-cycle arrest and apoptosis (Jones et al., 2008). The disease manifests in reduced proliferation and impaired migration of neural crest cells during development leading to malformations in the craniofacial structures (Dixon et al., 2006; Jones et al., 2008). Experiments in *Xenopus* embryos demonstrated that general growth defects occur upon stronger inhibition of Treacle (Calo et al., 2018). Future investigations of p53 status in tumors with high expression of Treacle should be conducted to identify if the p53 pathway is altered as a result of Treacle expression. Such investigations may provide further insight into how Treacle promotes tumorigenesis.

The importance of growth and proliferation in relation to cancer development was also assessed by Hu et al., 2022, studying tumor incidence upon injection of spheroids with or without Treacle knockout in the mammary fat pad of female nude mice. Mice injected with Treacle knockout cells showed significantly less tumor incidence, as well as lower frequency of tumor-initiating cells in comparison to the controls (Hu et al., 2022). Altogether, this could suggest a critical role of Treacle in regulation of cell proliferation, functioning as a pro-survival protein in tumorigenesis (Valdez et al., 2004; Dixon et al., 2006; Dai et al., 2016).

### 4 The Potential of Treacle as a Therapeutic Target

Accumulating evidence of Treacle overexpression in cancer raises the prospect of targeting Treacle as a novel anti-cancer strategy. The cellular response to Treacle silencing supports this approach (Wu et al., 2021), as does its role in both ribosome biogenesis and DNA repair.

Inhibiting ribosome biogenesis through Pol I targeting drugs has already been demonstrated to specifically kill cancer cells (Drygin et al., 2011; Bywater et al., 2012; Devlin et al., 2016). Clinical trials are being conducted and possibilities for combination treatment are also emerging (recently reviewed in Ferreira et al., 2020), underlining that ribosome biogenesis is a targetable pathway in anti-cancer treatment. For Treacle, the data available is very limited but a recent study showed that depletion of Treacle or treatment with chemotherapeutic agents in mice alone resulted in around 50% and 40% spheroid growth inhibition, respectively (Hu et al., 2022). Combination of Treacle knockout and treatment with chemotherapeutic agents resulted in approximately 75% inhibition of spheroid growth, demonstrating a promising synergistic effect of combination treatment (Hu et al., 2022).

DNA repair mechanisms have also become a widely used target in cancer treatment in recent years, with PARP inhibitors being a leading example (Rose et al., 2020). Drugs targeting DNA-repair pathways are often applied in combination with DNA-damaging agents or in certain genetic backgrounds leading to synthetic lethality (Topatana et al., 2020). Treacle’s function in rDNA repair and *in vitro* studies suggests that Treacle depletion/inhibition may also be advantageous under such conditions. Treacle was shown to lead to increased sensitivity to both IR, cisplatin, (Ciccia et al., 2014), and rDNA DSBs (Korsholm et al., 2019; Mooser et al., 2020). Another study investigated the recovery of normal salivary glands after radiation therapy, as damage to the salivary gland and consequent dysfunction is a common side-effect of radiation treatment in patients with head and neck cancer (Dirix et al., 2006; Weber et al., 2019). The authors found that Treacle protein level and phosphorylation of serine-792 were significantly increased in salivary gland progenitor cells isolated from radio-resistant rats after irradiation compared to radio-sensitive counterparts (Weber et al., 2019). These data support a potential Treacle-mediated radio-resistance mechanism and Treacle inhibition may therefore also have therapeutic advantages.

## 5 Discussion

**FIGURE 2:**
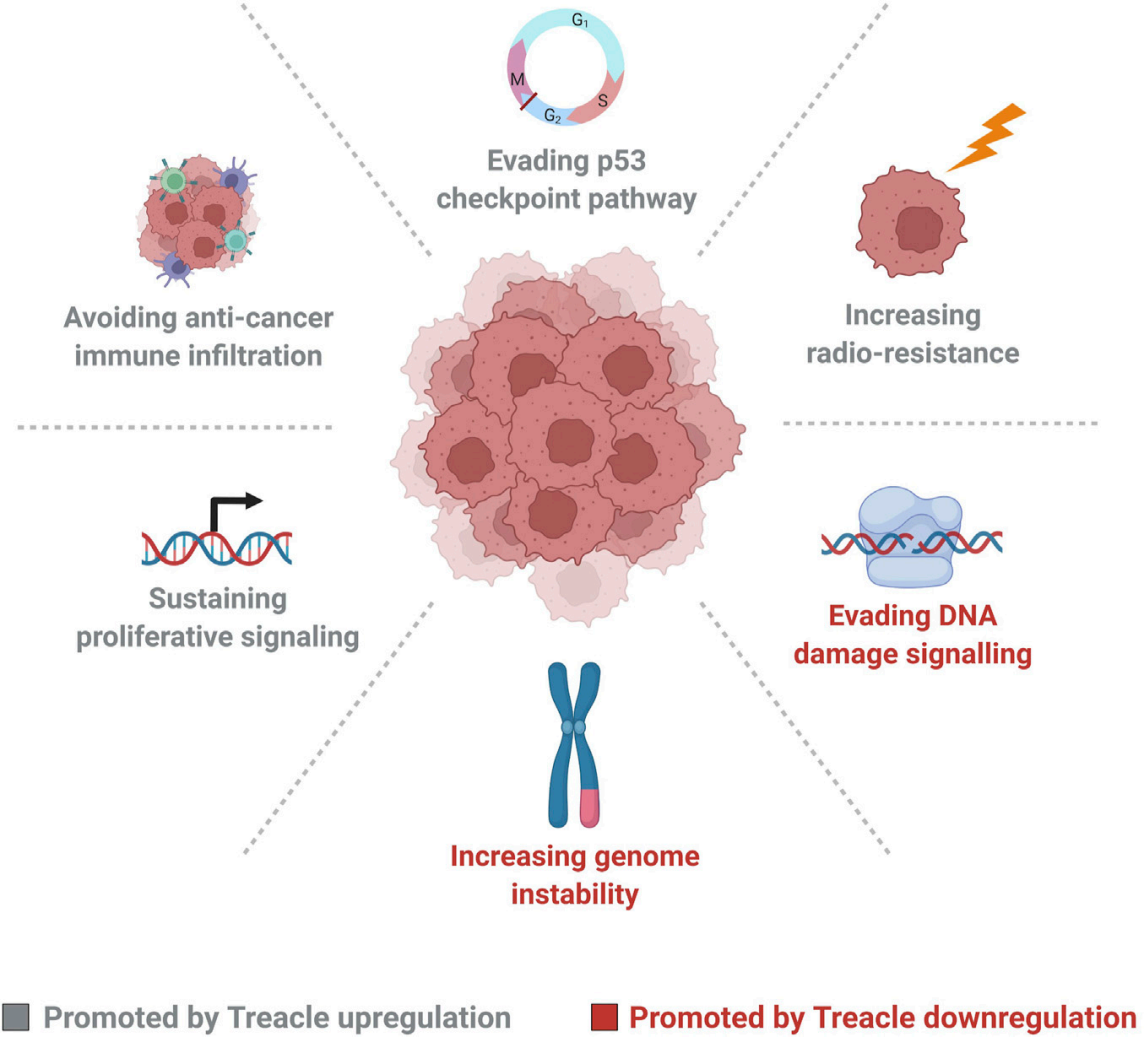
Up or downregulation of Treacle expression levels contribute to cancer initiation and progression through promotion of various cancer hallmarks. Cancer cells can benefit from Treacle downregulation, through loss of the nucleolar DNA-damage response, promoting genome instability, and evasion of DNA-damage signaling. Treacle upregulation may benefit cancer cells through sustained proliferative signaling, evasion of p53 checkpoint activation, avoidance of anti-cancer immune infiltration, and through increasing radio-resistance. Cancer hallmarks promoted by Treacle upregulation are marked in grey and hallmarks promoted by Treacle downregulation are marked in red. Figure created with BioRender.com.

There has been an increasing interest in the role of Treacle in cancer and its potential as a prognostic marker, as signified by the results presented in this perspective and recent studies examining the role of Treacle in cancer. In summary, they suggest that upregulation of Treacle expression promotes carcinogenesis, stimulates proliferation, survival, and possibly contributes to radio-resistance, albeit with variation across different cancer types (Figure 2). We observe significant upregulation of Treacle expression in 11 out of 14 different cancer types by analysis of paired normal and tumor data in agreement with other recently published studies (Wu et al., 2021; Gu et al., 2022; Hu et al., 2022). Interestingly, in uterine corpus endometrial carcinoma, where Treacle expression was not increased, the highest frequency of TCOF1 mutations were identified by Gu et al., 2022, possibly altering the properties of the expressed protein. Follow-up studies are needed to clarify if expression data accurately reflects protein levels and if Treacle is subjected to posttranslational modifications in cancer.

Our correlation analysis between Treacle expression and patient survival found significant correlation between high Treacle expression levels and poor survival in two (liver and renal) out of 18 cancer types. The correlation was also reported by Wu and others in a study specifically investigating human hepatocellular carcinoma, and in the pan-cancer analysis conducted by Gu et al. The number and types of cancers where Treacle expression correlates with patient outcome vary between studies, possibly due to the underlying data and the analysis method. In our analysis we separated patient groups using the median value, whereas Wu et al. and Hu et al. apply an “optimal cut-off” value. This approach takes the final endpoint into account and performs a retrospective separation of two patient groups. This approach generally finds more cancer types with statistically significant correlations between expression and patient outcome but is less suitable for stratification of patients. We therefore chose the median cut-off for analysis of Treacle as a potential prognostic biomarker.

Our analysis provides insights into how cancer cells balance the different functions of Treacle and suggests that elevated expression is favored in carcinogenesis (Figure 2). This is in agreement with nucleolar activity being broadly upregulated in cancers (Montanaro et al., 2008). Furthermore, other ribosome biogenesis factors, such as the Pol I transcription machinery and nucleophosmin, are also upregulated in cancer (Bywater et al., 2013; Chen et al., 2018). We found concomitant upregulation of Treacle and UBF, nucleolin, and Pol I in lung and stomach cancer whereas a variable degree of overlap was observed in other cancer types. Further investigations of co-regulation of ribosome biogenesis factors are needed to determine how increased expression is linked to ribosome biogenesis.

Wu et al. demonstrated a promoting function of Treacle in cancer initiation and progression through mechanisms related to proliferation, apoptosis, cell migration, transcription, and anti-cancer immune invasion in human hepatocellular carcinoma (Wu et al., 2021). Wu et al. demonstrated an inverse correlation between Treacle expression and CD8^+^ T cells, NK cells, and dendritic cells in HCC. A similar correlation between Treacle and immune infiltration by CD8^+^ T cells, CD4^+^ T cells, B cells, neutrophils, macrophages, and dendritic cells was also reported (Gu et al., 2022). Both studies point to a possible cancer-promoting role of Treacle through inhibition of immune infiltration, supporting a broader role of Treacle in cancer progression than previously anticipated (Figure 2).

The cellular addiction to ribosome biogenesis in cancer and the significant upregulation of Treacle across multiple types of cancer emphasize the importance of future research in cancer treatment approaches targeting Treacle. A major challenge lies in the development of an inhibitor targeting a protein where limited knowledge relating structure to function is available. Specific residues, however, have been identified facilitating protein-interactions, and peptides could potentially be developed that block the interaction between Treacle and its interaction partners. Whether this will provide sufficient inhibition of Treacle to kill cancer cells, however, remains uncertain.

The role of Treacle in DNA repair and the increased accumulation of DNA lesions observed after exposure to genotoxic stress, could present a therapeutic advantage. If a successful inhibitor could be developed, it holds the promise of inducing growth arrest and cell death, by abrogation of ribosome biogenesis, and at the same time prevent repair of lesions in rDNA. In combination with DNA-damaging agents, this potentially represents a powerful tool for cancer treatment.

## Data Availability

Publicly available datasets were analyzed in this study. This data can be found here: https://github.com/4ronB/tnmplot. Data was analyzed through the TNM plot described in Bartha and Győrffy, 2021. All scripts used in Bartha and Győrffy, 2021 can be found by following the link above.
